# A Historical Sketch of American Dental Patents: With Remarks

**Published:** 1852-04

**Authors:** Geo. W. Kendall


### ARTICLE V.

### *A Historical Sketch of American Dental Patents: with Remarks.* By GEO. W. KENDALL.

> "Tis a great fault in a chronologer
> To turn parasite: an absolute historian
> Should be in fear of none, neither should he
> Write any thing more than truth for friendship,
> Or else for hate."

ALTHOUGH many facts relative to the past history of the dental profession in the United States, have been recorded, one part of that history has been entirely neglected, and I have endeavored herein, by the collation of a mass of facts relating to patents granted for improvements in dentistry, to fill up this vacuum in our literature.

Until within the last fifteen years, most of the practitioners of dentistry, claimed only for it a position among the mechanical arts, and rested contented in the reputation of being *good mechanics*, and a proof of this was found in being a patentee.

The dark clouds which had so long obscured the dawn of dental science, have been partially dissipated by the rays of a more benignant planet, and now gives a foreshadowing of a glorious and brilliant future. A determination has been exhibited by many of our best and most scientific dentists, to elevate the standard of professional excellence, and to substantiate dentistry to be ranked as an important branch of the highest and holiest of sciences: the science of medicine.

Much has been done, and medical men generally recognize dentistry as a liberal profession. To show that this is well grounded, I will quote a few lines from "The American Journal of the Medical Sciences," April, 1851, which is as high medical authority as any in the world.

"American dentistry is doing every thing which the severest criticism can require, to establish its claims to the rank of a distinct specialty of medicine. The number and value of its periodicals: the rapid succession of new standard works; and the regular and steady increase of its collegiate foundations, challenge for our brethren in this department of the healing art, the highest admiration and praise."

To merit this, should be the aim of the dental profession, and to do it, we must cut off all compromise with quackery in any of its protean forms.

Medical ethics condemn nothing more surely, than patenting any thing connected with the practice of medicine, and this example has been followed by the American Society of Dental Surgeons. At the meeting held in 1847, "C. C. Allen offered the following preamble and resolution:

"*Whereas*, one of the objects of this society is to diffuse a knowledge of correct practice in surgical and mechanical dentistry among all practicing dentists, and to give all its members the benefits of all improvements in the science, thereby elevating it to the rank of a liberal profession: therefore,

"*Resolved*, That it is derogatory to the dignity of this society, to retain, as a member, any person who has secured, or shall hereafter secure to himself, by letters patent, any improvement in surgical or mechanical dentistry.

"*Resolved*, That no man shall be considered an honorable member of this society who has secrets in his profession, or refuses to impart any knowledge or information which he may possess, on the subject of surgical and mechanical dentistry or any collateral branches, to any other member."

Which were *unanimously adopted.*

The stand thus taken did much to elevate the profession in the eyes of medical men, but the next meeting, *although* in favor of the spirit of the resolutions, thought them premature, and they were *rescinded!*

This tampering with one of the worst forms of quackery, has cast a stain upon the American Society, which even the great number of really honorable members, will find difficult to remove.

We claim for dental science the rank of a liberal profession, between which and a *trade*, let us remember that the distinguishing feature is, that pecuniary gain is not its principal object. Liberality is a necessary attendant on great scientific discovery, and so freely has such knowledge been disseminated, that he who now attempts to secure to himself alone the benefits of such discovery, cuts the silver cord which bound him to the *truly* scientific and honorable, and exposes his own cupidity and mendacious pretension. In no other phase, are the attributes of quackery more definitely traced ; and here the inordinate selfishness of the quack stands out in bold relief; no high ambition glows in his breast, warming the ideal of his future into a hope of mental and moral greatness ; on the altar of mammon, of all claim to professional regard.

The profession of medicine, and even our own, young as it is, shows the history of quackery in the lives of men notorious, and frequently from this charlatanism, who have passed from their field of action, "unwept, unhonored and unsung," leaving a memorial behind, an abhorrent recollection.

In ninety-nine cases in one hundred, patents are secured, not for the profit that will accrue from the sale of the article patented, but for the purpose of creating an impression with the public to be felt in the office practice. It is a popular belief, that the commissioner of patents, with his assistants, is qualified to judge of an improvement in each and every department of knowledge. In the library of the patent office, are a great many books relating to those branches of art, in which patents are usually taken, but not half a dozen on dentistry proper. The applications for patents for articles which the corps can judge, are so numerous, that the examiners are scarcely able to give them a thorough examination, and for every application for an improvement in dentistry, unless the claim is so glaringly false as not to deceive any man, taken at random from the crowd, the patent is granted.

As illustrations of this, I would cite the cases of patents for "vertical extractors," three of which have been granted, and not one of them is of the slightest possible practical application, and yet almost identical in their mode of action. The key was patented five times ! once for nothing at all but a bend in the handle.

Another patent which was heralded as a wonderful improvement, and about which a great deal was said in Philadelphia at the time, by the inventor and his friends, was for making pin heads on the platina rivets of teeth. But to quote all valueless or filched patents, would be to leave few for further notice.

This laxity of examination at the patent office, together with the rigid laws of patents, make it almost impossible for a patentee to recover damages for infringement. Few dentists know any thing whatever of those laws, therefore apply to a patent agent who knows as little about dentistry as the examiner of patents, and the claim and specification is prepared in a *legal* form by him. In consequence, the inventor claims too much, or describes not enough. He may claim a number of improvements, of which some small and unimportant one is not his own, and then all action for infringement is at an end.

Before an inventor shall receive a patent, he shall "deliver a written inscription of his invention, of the manner of making, compounding, using and constructing the same, in such clear, full and exact terms, as to enable any person, skilled in the art or science to which it pertains, to make, construct, compound or use the same." Government grants him a monopoly of his invention for a term of years, and, in return for this protection, demands that it shall be made public property at the end of the term. That this end may be accomplished, the government requires such a description as will enable one skilled in the particular art, to make the thing invented, by simply following the said description.

I will now proceed to point out a few legal points that present themselves.

The "inventor must first swear that he believes himself to be the first and original inventor." The first point under the United States law, is that of novelty, which relates not merely to previous inventions in this country, but any where in the world. No matter how secretly it had been used in this country prior to the application for a patent, proof of such use will be sufficient to invalidate the patent. It is not enough, however, to debar the patent, to show that it had been used in a foreign country unless it had been patented or described in some public work. The courts do not require that the desription should fully answer the purpose of specification, but only that it should serve as a direction for making or doing the thing to which the description related.

The consideration upon which a patent is granted, is novelty of every material thing, process or part, of the invention that is represented as a substantial part thereof: this consideration is an entirety, and, consequently, if any part of it fails, the whole patent is invalid. Whatever is covered by the claim of the patentee as his invention, must be taken as a part of the claim; if it turns out that any thing claimed is not new, however small or unimportant such asserted invention may be; the patent is void, and prosecution for infringement can be sustained. To show the extent to which this is carried by the courts, the case of Campion *vs.* Buryon, may be cited, in which a patent taken out for "making sail-cloth without starch," was declared void, as the true improvement consisted in a peculiar texture or twisting (described in the specification) by which he was enabled to manufacture cloth without starch, but starch had been excluded before. If a patent contains more than is necessary for the production of the described effect, and the addition was made for the purpose of evasion or deceiving the public, or if the whole specification fails to produce the described effect, the patent is void, and an infringement will not have taken place unless the patent can be successfully practiced by following the specification, an infringement being a copy made after, and agreeing with the description laid down, and if the patent does not fully describe any thing essential to the making or doing of the thing patented, there will be no infringement by the first invention of processes, which the patentee has withheld from the public. (See Webster, Pat. Cas., 167.) Nor will it be a violation of the inventor's rights, to make a machine, or composition, for the purpose of ascertaining its sufficiency to produce the desired effect, or to ascertain the truth and verity of the specification.

THOMAS BRUFF, Senr., of Maryland, June 27, 1797. This is the first patent granted in the United States for an improvement in dentistry, and somewhat curious as a relic of a dentist whose name has never appeared in any of our numerous dental publications. From the fact that James Gardette's name appears as one of the witnesses, it is probable that he was a dentist of some ability. The patent was granted for a "perpendicular extractor."

"This instrument has a double claw, with a joint near the middle, and a spring to draw them together when set on the tooth. It has a fulcrum with two branches, one to answer as a handle for the left hand, to keep it on the adjacent tooth; the other, having a hook in the upper part of the end, serves as a guide and support to the lever which passes through it, and through the staple at the extreme end, and the point through the eye of the claw; under the fulcrum is a crooked cap (sliding on with a screw head, and turning on a pin) made to shift to suit the side and shape of the jaw.

"There is another cap made long, designed to rest on the front and back teeth equally, having a hole through the middle large enough to draw the claw through. The burr has a handle nearly like the common key instrument. It has a crooked blade near the extremity, to raise it perpendicular from the fulcrum; when turned by the handle, it passes through the hook of the fulcrum to the shoulder, which is about two inches from the handle, the point being small and round, passes through the eye of the claw, (which is sharp at the top,) and by a turn on the back of the blade, brings out the teeth in a perpendicular direction." Signed, THOMAS BRUFF, Senr.

Witnesses: JAS. GARDETTE and RD. CENAF.

E. BRYAN, July 13, 1829. Vertical extractor, in which the claim is for making the claw to move on swivels. Mr. B. is better known as the biographer of Mr. Greenwood.

J. W. RUTHERFORD, Albany, New York, January 4, 1831. Vertical tooth extractor: no peculiarity either better or worse.

MOSES R. HANSON, Bangor, Maine, March 12, 1836. This is a forcep well fitted to the class of teeth to be extracted, with an attachment to rest on other teeth as a fulcrum.

DANIEL H. DICKEY, Boston, August 10, 1838. Extractor. This is so extremely *ridiculous* as to deserve notice. It consists of two claws so jointed that a wedge forced down between the ends opposite the tooth by means of a screw, will cause them to take a firm hold. To apply it, a plate with a hinged joint and hole, just large enough to admit the claws up to the joint, is laid along the adjoining teeth, the claws slipped through the hole on to the teeth; the wedge driven down by means of a screw, the end of the jointed plate depressed, while the tooth is raised *vertically.*

CORNELIUS ADDLE, Winthrop, Maine, July 13, 1833. A key, in which the only difference is, that the stem had a bend in it, by which means it was easier of application.

JOHN MCCONNELL, Philadelphia, September 29, 1839. A complicated key, in which one hook answers for all sizes and classes of teeth.

MOSES J. HILL, Bloomfield, Indiana, June 7, 1841. A key in which a friction roller constitutes the bearing part of the bolster.

J. W. BAKER & W. W. RILEY, Columbus, Ohio, Nov. 8, 1845. A combination of the key, with forcep handles, but the action is the same as the key. For those who prefer the key to the forceps it may be of some advantage if they should have a patient prejudiced against the use of the key.

ENOCH OSGOOD, Bangor, Maine, Jan. 9, 1849. "My invention is a compound fulcrum, partly concave and partly convex, of which the former rests on the surrounding teeth, and the latter on the tooth to be extracted."

HIRAM TODD, Columbus, Ohio. Cylinder key.

The claw is fixed to a rod which passes through the hollow stem, connecting with a rack and pinion, worked by hooks on either side. It is easier of adjustment, and the same extracting power.

CHARLES H. DUBBS, Natchez, October 17, 1848. Screw forcep. A combination of the notches on the shaft of the screw, with the catch of the click; by means of which the screw affords additional power in extracting roots of teeth.

The simple combination of screw and forceps, is due to Dr. S. P. Hullihen, of Wheeling, Va. (See Am. Journal of Dental Science, June, 1844, vol. —.) C. H. Dubs added the catch for holding the screw firmly, and patented it as above. John D. Chevalier produced the same effect, by a slightly different mode, which Dubs insists is an infringement. A violent controversy in the dental journals, as to priority of invention, ended as it begun.

Chevalier still advertises his "improved Hullihen screw forceps," so it is probable, that Mr. Dubs has not thought the ground sufficient to sustain an action.

"His mode of disposing of his improvement, is to sell the right of making, and individually using it, to any dentist, as a *life interest*, for three dollars." In three years his monopoly will be at an end, a rather short *life* interest.

KIRBY SPENCER, Athens, Georgia, October 17, 1848. Dentist's drill.

It is a tapered tube, in which are concealed the steel works which combine the endless chain, pulley, pinion and piston. The movement is like that of a syringe. It is a very handsome instrument, and very useful, but so complicated as to render it too expensive.

L. D. WALTER, Fort Plain, New York, May 15, 1847.—Dentist's drill.

"A combination of the main-spring, pulley and cord."

LEMUEL MERITT AND S. ROGERS, New York, December 27, 1815.—Relieving tooth-ache by steam.

JAMES UTLEY, ———, November 5, 1817.—Tooth-ache remedy.

HORACE H. HAYDEN, Baltimore, Md. February 11, 1824.—Preventing caries of the human teeth.—*Claim.*

"The exclusive privilege of using and vending the empyrheumatic oil (tar or balsam) and acid, obtained by the distillation of wood, which oil or acid, when properly modified, proportioned and applied, is used for the purpose of counteracting decay in the human teeth, and the diseases consequent thereto, and to the human mouth.

"By a daily use of it for two years, I have proved it to be a sovereign remedy for the diseases mentioned. Its specific qualities are owing to the antiseptic property, which counteracts the caries in the teeth, allays the pain and irritability of the vessels of the teeth and mouth, lessens the morbid sensibilities, and arouses and restores a healthy action. For deep caries and great sensibility of the parts, I apply the pyroligneous oil, with a very small proportion of the acid, aromatised with oil of cinnamon, cloves or other essential oil, to the cavity of the diseased tooth, on a deposit of cotton. This is repeated several times, as occasion may require. To correct a general disposition in the teeth to decay, for scorbutic, ulcerated or other diseased state of the gums, I use a solution of acid and oil in three parts of water, as a gargle three times a day."

This patent has been entirely overlooked by Dr. Hayden's biographers, and show what errors great and good men may fall into, but Dr. H. nobly and well atoned for an error which, at that day, was comparatively venial, by the course he assumed during the last years of his life.

SAMUEL PENNINGTON, Mt. Pleasant, Ohio, July 30, 1829. Specific for tooth-ache. Composed of "whiskey, French brandy, spirits of turpentine, tar and Indian turnip."

L. S. PARMLY, New York, June 17, 1820.—"Composition for preserving the teeth."

This is now known as "Parmly's tooth polisher." The record at the patent office being destroyed by fire in 1836, I am unable to give the ingredients.

CHARLES NEWTON, New York, September 25, 1825.—"Apparatus to keep free from saliva." Record lost.

WM. R. EAGLESTON, Baltimore, October 4, 1817.—"Setting natural and artificial teeth." Record lost.

CHARLES GRAHAM, New York, March 9, 1822.—Artificial teeth. Record lost.

ELIJAH A. BIGELOW, Brandon, Vt., March 8, 1827.—Engrafting teeth. Record lost.

ANTHONY PLANTON, Philadelphia, April 5, 1828.—Mineral teeth without platina.

"The inventor says, that necessity, which is indeed the mother of invention, suggests this improvement: platina being so scarce that it could not be procured readily. A small hole is made through the tooth at the time of moulding, which, after baking, is filled up with a wire, and solder is melted on both ends of it: or a hole is made in the tooth, but not passing entirely through; into which, the wire is introduced and solder fused around it. Teeth made in this manner, will never break or separate from their connections, and time will show that this improvement brings artificial teeth to their perfection." Most of the English teeth of the present day are made in a similar manner, except that the hole is bushed with platina.

THOMAS R. VANDERSLICE, Philadelphia, January 5, 1831.—Artificial teeth.

"Instead of applying a metallic plate on the back part of the tooth in the usual manner, the tooth is moulded with a groove in the back part, into which the plate will fit, and secured to platina wires set in the usual manner. To form the tooth in the ordinary mould, a small piece of ivory or other hard substance, with holes drilled to receive the wires, may be introduced."

Were we disposed to be fastidiously nice about the novelty of this, the old French teeth might be adduced, in which a groove was left for the reception of a wire, which was soldered to the platina cramps, and could be cut down flush with the porcelain, as in the above.

SAMUEL G. CHAMBERLIN, Philadelphia, February 11, 1831.—Artificial teeth.

"Pieces of wire are inserted in the body of the tooth, previous to its being baked, projecting from the top, and to them the gold plate is to be soldered, by which the tooth is to be attached. I claim the glazing of the inside of the tooth, and polishing of the gold plate, so that the tooth may not absorb moisture, and no unpleasant roughness present to the tongue; the manner of fixing the tooth by means of platina and gold plate. This would differ from all others in this, that there is no interstice between the tooth and plate for food or moisture to collect."

It was probably necessary to solder the plate, (strap or stay,) after fitting to the plate, and before soldering to the main plate.

DANIEL HARRINGTON, Philadelphia, December 10, 1840.

This improvement consisted in using screw or pin heads, on the platina wires, or a staple, instead of the chisel or flattened form, inserted.

HENRY LAWRENCE, Philadelphia, May, 1849.—Artificial pivot teeth.

"I make a headed screw of gold or other suitable metal, of a size sufficient to hold the pivot tooth firmly. My tooth is of the same size and shape as the ordinary pivot tooth, but with a hole entirely through, terminating in a countersink, to receive the head of the screw. The tooth being fitted, and the canal of the root prepared to receive the screw firmly up against the root." The claim is for "a tooth with an aperture clear through it, terminating in a proper bearing for a screw-head."

GEO. E. MURRAY, Philadelphia, December 4, 1849.—Artificial teeth.

After stating that the ordinary plate teeth are objectionable, for the following reasons, viz. if ever so well fitted, there will be a space between the strap and the tooth, the fastening is at a point above the edge of the tooth, allowing a leverage power. The rivets are frequently ground out, and in meeting, being in great danger of breaking. He describes his as follows: "A piece of metal of proper size, is taken, and the edges bent up: this plate is placed in a mould with the flanches inward, and the porcelain paste filled in as usual, and the plate will, by means of the flanches, become securely fastened to the tooth. Various variations of the plate may be made, for example, a flanche may project from the center; or the inner edge of the flanches be made thicker than at any other point: they may be bent at right or oblique angles," etc. The claim is for "an artificial tooth, having a plate combined therewith."

For very many cases, it is the best article made, and is peculiarly adapted to molar teeth. The same tooth is described by M. Desirabode, in his "Science and Art of the Dentist," translated by C. A. Harris, M. D., for the Am. Lib. of Dental

Science," in 1847, I find the following, page 411. After describing various modes of inserting cramps, he says, "to obviate these inconveniences, we have fixed in the tooth before burning, not simply cramps, but a piece of metal forming at once the cramps and the shaft by which the tooth is to be fixed to the plate.

"This fixture is composed of a central shaft, from each side of which go off in the form of wings, little cramps, which pass into the paste and disappear."

This publication invalidates the patent.

W. WILSHIN RILEY, Columbus, Ohio, Nov. 18, 1851.—Artificial teeth.

"I claim the concave base, and the insertion of the platina in an oblique direction, and mode of attaching them to the plates without stays." The platinas entered the tooth at the lower internal edge in an oblique direction, so that when fitted, they rested against the plate, and were soldered to it. The tooth was rather thicker than usual, especially the six front ones, so that the base reached *over* the alveolar ridge, forming the concavity spoken of, and brought a greater surface of porcelain in contact with the plate, than in any other tooth. From some cause, the manner of inserting the wires has been abandoned, and now are inserted little *flat* pieces of platina, in about the same place as are the platina pins in our ordinary tooth.

Between these slips, after the tooth is fitted to its place, is passed flat pieces of gold, which is soldered to them and the plate. A continuous lining strap may be made very easily and neatly with these teeth.

JAMES CAMERON, Philadelphia, July 30, 1840.—Articulating instrument.

A perpendicular rod, "fixed in a base, with two sliding shafts similar to the lamp stand of chemical laboratories. A rod bearing a plate designed to support the upper jaw plate, passes through a hole in the vertical rod, and is held at any point by a thumb screw; below, is another plate, shaped something like the under jaw, (designed to support the lower jaw plate and draft,) which is moved up and down on the rod at pleasure. It is attached to the tube which slides on the rod, by a hinge, which opens up and down, and is regulated by means of a nut and screw.

The plates with the drafts, are fastened to the frame by screws, and the different parts of the instrument adjusted. The upper jaw is fixed like the human jaw, except, that it may be moved backward and forward: the lower jaw moves upon the hinge like the human jaw, and also up and down on the vertical rod. This is the best articulator known to me, although somewhat complicated, so much so, indeed, that it is difficult to understand a description without drawings.

The instrument as patented, excepting the foot in which the rod is fixed, was *invented by Daniel Neall, Sr.*, of Philadelphia, several years before Cameron secured his patent, and the same instrument was on sale in that city in 1838.

Neall was one of the pioneers of block work in this country, and a man of great ingenuity. He invented the "Vertical Printing Press," the best of its day, for which he received a gold medal from the Franklin Institute.

To him, also, is due the credit of perfecting drafts or models for the construction of artificial teeth, and a description of which will appear in a more appropriate place than this article, the same never having been noticed by previous writers.

DANIEL T. EVANS, Philadelphia, August 28, 1840.—An improved mouth mould, which being filled with wax, is taken of both jaws at once, by closing them on the wax. This impression is to be placed in a hinged articulator, and filled with plaster. The mould is made without a dividing plate, so as to allow the jaws to lap in taking the impression.

JOHN ALLEN, Cincinnati, Ohio, Dec. 16, 1845.—Restoring the contour of the face.

"I claim the manner of restoring hollow cheeks, by means of metallic bulbs, constructed in the manner set forth, or by any other substance between the jaw-bone and the cheek."

The restoration of parts about the mouth, lost by accident, has long since been accomplished; and alveolar absorption is replaced by block work. But this is not the object of the present invention, which is to fill out the sunken cheeks which are so frequent an attendant on advancing age. But, to be brief, I will insert a few extracts from a letter recently placed in my hands, addressed to a dentist, which gives the original idea, and the gist of the whole matter, dated

*Dear Sir*, JUNE, 1848.

I take the liberty of addressing you on a subject, the accomplishment of which seems to me desirable.

It is relative to the insertion of a set of teeth. Most of my lower teeth, and the very hindermost of my upper teeth are lost, and thereby gives way for the cheek to follow them, so that my cheeks are sunk in, rendering my face, if not deformed, at least meagre and visage like. * * * The upper front teeth cast out a little, and the upper double teeth, and one or two nearest them on each side, cast inward, so that when the mouth is naturally closed, the upper teeth fall inside of the lower teeth and gums. * * * *

I have to remark, that between the socket bone of the eye, and the prominent part of the upper gum, there is a hollow, in which the finger of a man might lie and fill it on a level only with that part of the gum which is swelled out with the body of the tooth. Sir, I am anxious to know, could this hollow part be filled up, so as to push out the skin.

I would suggest that the lower teeth which are to be inserted, be so prepared that they be outside of the upper teeth, and long enough to shoot over the upper gum, and come as far as the hollow part before described, passing just over it, effecting the thing desired. * * * * *

Sir, I would suggest that your trade is one of improving the creation, and did you ever remark that the only thing that mingles old age, though sometimes even the young countenance, is wrinkles under the eye, and a loosening of the skin. Now, if you put your finger on the temple of your eye, and push the skin towards the top of the ear, the countenance changes for the better; youth flushing in the countenance, and the wrinkles completely gone. Perhaps an operation to effect this, would not be painful, and if the skin when straightened, could be made fast, the thing were done.

Let me hear much from you, and tell me, could you effect what I desire. Seal your letter well.

Yours, respectfully, J—— G——.

Some time after, the writer of the above, called on Messrs. Cook & Hunter, dentists, for an operation substantially as described. They would do nothing but put in a set of lower teeth, articulating with the upper, but this would not satisfy him. They filled the *hollow* with cotton, pushing out the cheek, and that was just what he wanted, but these dentists were pleased to call it ridiculous and absurd, and refused to make a permanent fixture to produce the same effect.

Mr. G. then called on Dr. A., who soon devised the elegant fixture, for which his patent was granted, and which accomplished one of the ultimata proposed by Mr. G. The other is more difficult, but equally necessary, for when the plump cheeks of youthful beauty once lost, are again restored, their contrast with a wrinkled brow and shrivelled lips and sallow skin, makes ruin more appalling, especially if it is attended by a hollow, sepulchral voice, as are most of these attempts at restoration "of pristine beauty."

"Beauty is a doubtful good, a glass, a flower,
Lost, faded, broken, dead within an hour;
And beauty blemished once, forever's lost,
In spite of physic, painting, pain and cost."

At the meeting of the American Society, held in August, 1845, Dr. Allen presented his invention, and received the approval of that body, a gold medal, and five volumes of the "American Journal of Dental Science." Four months after that time, his patent was issued.

At the meeting of the society in 1847, a vote of censure was passed on Dr. John Allen, for having procured a patent *after* having freely offered the benefits of the invention to any member of the society, and afterwards demanding a per centage for using it.

The editor of the Dental Recorder, vol. 2, page 120, says, Dr. Allen freely granted "us the privilege of using it in our practice; but this was after we had assured him that we had seen the same principle adopted for the same purpose, some years before his patent was granted, and that we should not hesitate to make use of it whenever we saw fit."

J. SMITH DODGE, New York, March 13, 1844.

"I claim the mode of inserting artificial teeth by inclosing a metallic tube within the wooden plug or cylinder, usually employed in fixing artificial teeth."

"I make the tube of gold or other suitable metal, of such length and size as may be required: with a cap or stopping at one end, to prevent moisture from entering the cavity, causing internal decay; or without the cap leaving a free opening through its entire length."

F. HAMILTON CLARK, New York, Feb. 13, 1849.—Securing pivot teeth.

"I make an opening in the root, in the usual manner, being careful to make it perfectly smooth, round, and of an equal size, from top to bottom. Into this opening, I insert a cylinder of gold, or other metal, made to fit it with great accuracy, but not so closely as to require much force to introduce it.

"This cylinder has a bottom of a spherical form, with a hole in its center, for the purpose of allowing a screw to pass through it into the center of the root, which is pierced and tapped to receive it. A flange encircles the outer end of the cylinder, for the purpose of retaining a filling inserted in the end of the root, when decayed. It has also a bar soldered across its inner side, about midway of its length, to hold the metallic pivot to which the tooth is attached, and occupies about one-fifth of the diameter of the tube. The screw which passes through the bottom of the tube and holds it permanently in its place, has a head fitted to the bottom, which being rounded, allows the screw to follow the opening made to receive it, although it be not parallel with the bed of the cylinder; hence it is perforated lengthwise, to allow the pus to escape.

"The pivot is made of a wire drawn to the exact size of the interior of the cylinder. The end to be introduced, is to be split with a fine saw, nearly to the tooth, one side is filed flat to allow it to pass the *bar* against which it rests. A tooth set in this manner is easily removed, for the purpose of cleansing, and may be made very firm by inserting a very thin slip of wood in the cleft of the pivot.

"I claim the mode and manner of securing the cylinder in its place, as set forth."

This is a very neat mode of inserting pivot teeth, but it requires such delicate manipulation, as to greatly retard its general introduction.

The mode recommended by Professor Harris, in his Dental Dictionary, of cutting the screw on the outside of the cylinder, is much easier, and quite as good.

Desirabode describes, page 469, a mode of inserting pivot teeth with a screw; a mixture of Harris' and Lawrence's mode.

M. LEVETT and H. DAVIS, New York, Sept. 18, 1847.

"An invention for concealing the metal work used in the insertion of artificial teeth. It was done by means of a varnish composed of equal parts of shellac and linseed oil, with a little spirits turpentine, ground up with bismuth, vermilion and cobalt, in sufficient quantities to give the desired color. This is softened with spirits of turpentine, and applied over the surface of the plate and clasps with a brush." This was quite tough, and a plate to which it was applied, could be bent back and forth without detriment, but it could not withstand the action of fluids of the mouth.

Some kind of enamel was then prepared by Levett, and "rights" were advertised for sale. I am not certain that he sold *patent* rights to use it, or claimed it as a part of his patent, but presume that he was too well acquainted with the machinery of the law, not to evade this point. The exclusive right to this city, was purchased by Dr. John Allen, and used by him two years ago, being made a *feature* in his practice; but he found that the gum had not strength enough in the form he got it, to withstand the force which was applied to it in the mouth. In using it the teeth were mounted on gold in the usual manner, and the gum applied beneath and around them, and fused.

For a more extended notice, see C. T. Cushman's article, American Journal Dental Science.

JOHN ALLEN, Cincinnati, December 23, 1851.

"I claim, as my invention, a new mode of setting mineral teeth on metallic plates, by means of a fusible silicious cement, which forms an artificial gum, and which also unites teeth to each other, and to the plates on which they are set. I also claim to be the inventor of the said cement or compound, a full and exact description of which is herein given. I also claim the combination of asbestos with plaster of Paris, for covering the teeth and plates for the purpose of sustaining them in their proper position, while the cement is being fused."

"The cement may be formed of any of the known fluxes, combined with silex, wedgewood and asbestos, intermixed with gold and platinum scraps, which form a metallic union with the plate. The compound which I prefer, is composed of *silex*, 2 oz., *white or flint glass* 2 oz., *borax* 1 oz., *wedgewood* 1½ oz., *asbestos* 2 drachms, *felspar* 2 drachms, *kaolin clay* 1 drachm. This should be intermixed or underlaid with gold and platinum scraps. The *gum color* consists of felspar ½ oz., white glass 1 oz., oxide of gold 1½ grains; mix, moisten and apply with a brush.

"The plates are constructed and the teeth arranged thereon in the usual way; I then apply the cement in a plastic state upon the outside, between and around the base of the teeth, so as to form an artificial gum; the teeth and gum are then covered with a mixture of asbestos and plaster, and the wax removed from the inside of the teeth, and the cement is applied thereupon, and also on the plate, so as to fill up all the interstices. When dry, the piece is put into a furnace, and heated sufficiently to fuse the cement, when it is withdrawn and cooled slowly. The plaster mixture is then removed, a gum color applied, and the work again placed in the furnace, and fused, as before, by which means the metallic back plates, solder and blow pipe are dispensed with, although back plates may be attached to the teeth if desired."

The first public announcement of this discovery was made in the "Dental Register," which appeared about the first of May, 1851, about two weeks after the filing of his caveat.

The first and most important claim—that of attaching teeth to plates by other than metallic connections, is taken bodily from a French work entitled "Traite de la Partie Mechanique de l'Art Chirurgie Dentiste, par C. F. Delabarre." Paris, 1820, page 228. A very correct translation of which appeared in Fitch's Dental Surgery, second edition, 1835, page 271. Unfortunately for the position assumed in the "Register," he has found that this mode of fastening is not sufficiently strong for the mouth, and now the teeth are soldered to the plates, (which must be platina, as the 22 *carat gold* will warp when subjected to the requisite heat) and the gum filled in and brought up. From three to six heats are necessary in large cases.

Delabarre describes (page 240) a mode of setting teeth by soldering them to the plate, by platina wires, adjusting them in the mouth and filling in the base which shapes the gum, which is colored in a subsequent heat. It is also briefly described in Desirabode's "Science and Art of the Dentist." (Am. Lib. Dental Science, edition) translated by Prof. Harris, page 456, and in Harris' Dental Dictionary.

If Dr. Allen had not seen any of these works, it argues badly for his acquaintance with dental literature, and his knowledge of what has already been done in mechanical dentistry.

There is now in the museum of the Baltimore College, a double set of teeth, mounted in this manner, made nineteen or twenty years ago. A set of teeth soldered to platina plates, with the backs covered with a gum colored enamel, the arch in each jaw continuous, and a set mounted on silver in the usual manner, viz. the full jaw *completed* in one piece, is placed on the plate, and soldered by means of the usual backs, were sent to the World's Fair, by Wm. M. Hunter, of this city, 13 months ago. One or both of these sets was described to Dr. Allen, months before Allen had produced a single specimen of his patent or present style. So much for the *novelty.*

The second claim is defective also. If the ingredients are simply mixed and applied, the water of crystallization in the borax will be driven off so rapidly as to destroy the symmetry of the work. The mode of preparing enamels are so various, that it would be difficult to say in what precise manner this one should be made. As he says, just below, in reference to his gum, *mix, moisten and apply*, the natural inference is, that this is to be done in the same manner.

The patent is wholly invalid, and there is not the slightest possibility of the inventor sustaining a suit for infringement.

The Mississippi Valley Association, with the exception of one individual, swallowed *the improvement*, patent, secret and all, recommending it to the profession without knowing a single ingredient of the compound, or any thing whatever of the necessary manipulation!

> "All, with one consent, praise new-born gauds,
> Though they are made and moulded of things past."

Dr. A. submitted specimens to the American Society, at its last meeting, for its consideration, and said that he was "opposed to taking out patents for professional improvements, and although a caveat had been filed in the patent office for this improvement, it might be there for ever. This promise has been literally adhered to, and the caveat is *still* in the archives of the office.

Notwithstanding this liberal promise, the society could do nothing for him except giving thanks for the exhibition of specimens.

This patent is demonstrated in the "Ohio College of Dental Surgery," the students of which are *peculiarly* favored; and it is held out as a card of inducement to students.

ASA HILL and EMANUEL G. BLACKMAN, Norwalk, Conn., February 13, 1849.

"We claim the combination of gutta percha, as a base, with such other earthy mineral and metallic substances, as will shorten it, and render it less tenacious, harden it, and render it fit for a useful filling, and give it the *desired color*, without any noxious quality, or destroying its plasticity when heated, and the application of such compound substance to the filling of carious, hollow and defective teeth.

"This article is made by taking the gutta percha of commerce, and freeing it from its impurities and coloring matter, by boiling and working it, or by maceration, and combining with it when sufficiently heated with a dry heat to render it plastic, about two parts quick lime and one part each of quartz and felspar, all reduced to an impalpable powder, and may be varied in color by the addition of other earthy mineral or metallic substances, and slightly in hardness, by adding the filings of any of the metals used in filling teeth.

"It is entirely innoxious, becomes plastic at a moderate heat, hardens again when applied, and when reduced to the heat of the body, adhering firmly to the cavity, and is sufficiently hard and permanent for mastication."

When the American Society of Dental Surgeons fulminated its anathema against the use of amalgam, some *substitute*, easier of application than gold, and yet not liable to the charges brought against amalgam, become a great desideratum.

Soon after, the discovery of such an article was announced, and it appeared in the market, as "Hill's Soft Stopping," put up very neatly in small bottles labeled with "directions for use," *a la* Brandreth's pills, and sold at the moderate price of $15 per bottle. Although the inventor flourished his professional credentials (being a graduate of the Baltimore College and a member of the American Society) over his bantling, quackery was so apparent in his heraldry, that but little attention was paid to it at first by the profession. In the June number of the New York Dental Recorder, may be found a circular setting forth the claims of the inventor, and in August number, he still further sounds the praise of the invention, and takes the field in defence of patents for professional improvements.

Now A. Hill, D. D. S., etc. claims an equality of professional standing with those men who have freely given to the profession improvements infinitely more valuable than his, and at the same time assumes the position, and gets the profits, which are about 6000 per cent. of the nostrum vender! two positions wholly incompatible.

He has chosen, perhaps wisely, to place his invention under the protection of the *patent law;* by so doing he takes ground with the thousand and one nostrum makers, is exposed to captious ingenuity, and the same legal, lean, wire-drawn, hair-splitting distinctions will be applied to him, as to them.

I shall endeavor to show, that by his attempt to reconcile professional dignity with an absorbing love of gain, he has forfeited all claim upon the profession; that his conception of the character of a scientific professional gentleman, is far more defective than is his knowledge of the practical application of the law of patents, which has failed to throw any safeguard whatever around him.

His assertion that the principle of rewards of scientific improvement as shown in the medals of scientific societies, the diplomas of colleges, and copy-rights to authors, is neither identical, or even in the remotest degree similar to that of the patent system, is too absurd to admit of argument.

He says, that it (the stopping) is difficult to prepare, and with a recipe and full instructions, it would be almost impossible to prepare the article, and assigns this as a reason why he *should take out a patent.* At the meeting of the American Society in 1848, he presented the claims of his compound, but declined giving the ingredients; but a very short time afterwards, (if he had not already,) sent to the patent office the above claim and specification, a copy of which may be had from that office by any one wishing it for sixty-six cents; and he says, under oath too, that that is the best recipe and mode of preparation known to him.

The color of gold has always been an objection to its use, especially in front teeth, and a substance of the same color as the tooth, which would protect it from decay, resisting perfectly the chemical action to which it is exposed, is, and always has been, a desideratum.

The inventor says that this is such an article. He also claims in his patent, it is sufficiently hard and permanent for mastication, a point which he abandons. (See Recorder, vol 5, page 259.)

The color of the specimens sent to this city by his agents, Jones, White & Co., are so dark as to be more offensive to the eye than highly polished gold fillings.

The failure to accomplish these two points is sufficient in itself to invalidate the patent.

Any one who chooses, can make an article identical with that sent to this market, sell it when and where he pleases, and still the patentee could not sustain an action for infringement.

I consider it the best material in use for filling teeth too much decayed to be filled with gold, and as a *temporary* filling for sensitive teeth. It is also absolutely necessary to those dentists who are unable to put a solid gold filling into a difficult cavity; for a filling of it, well put in, is far preferable to a bad gold plug, which is worse than none. Its use is suggested in case where the nerve is nearly exposed, as it is more easily applied than asbestos. In the Recorder, vol. 6, page 18, the inventor describes an application of it to the insertion of artificial teeth dispensing with the use of plates entirely.

MATT. S. FOSTER, Trenton, N. J., Nov. 12, 1842.

"The nature of my invention consists in soldering a strip of metal of the thickness of a half-worn sixpence, and one-eighth of an inch in width, (more or less,) to the outer edges of the superior and inferior plates, designed to have incorruptible teeth fastened upon them; which teeth, are usually made in three sections, but varying to fourteen. The flange is intended to give greater strength to the arch, and prevent the introduction of secretions between the joints."

This was the first *publication* of what is now known as running or banding, although it was frequently used by block-workmen in order to conceal imperfections of filling, &c.

GEORGE STEWART, Phil. July 3d, 1847.

"Dental lever joint spring. I claim the manner of forming the spring joint and arms, by coiling the middle of the wire so as to constitute the spring joint, and extending the outer ends thereof so as in part to constitute the elastic arms of the lever, in combination with a check plate."

An elastic wire is wound in the middle around a mandrel, about one-fourth of an inch in diameter, about three times. When the extremities of the wire are made to approach each other, the convolutions are diminished in diameter, and have a tendency to throw the arms apart. Through the spiral is placed a wire terminating on either end in a flat head of the same diameter as the spiral. Small spiral springs are passed over the arms and soldered at the outer extremities, so as to constitute one piece. The check plate alluded to, is intended to prevent the spring from being unduly bent, and is connected with the lower jaw.

This is a very pretty looking spring, and in some cases preferable to the usual form of spiral, but the general plan was described and figured by *Delabarre*, in the edition of his work published in 1820, page 444. Figures 184, 189.

The first publication of any thing relative to *chambered plates*, was the patent.

ALFRED RIGGS, New York, July 3, 1840.

He struck a plate to fit the mouth accurately, and perforated a portion of the surface resting on the palatine vault, with small holes. Over this, a plate was struck, forming a chamber about one line in depth at the center, vanishing to the alveolar ridge, and soldered firmly to it. The chamber thus formed, was at times divided into sections. In practice, it was found that the gum was drawn down into these holes, causing such painful inflammation, that the plate could not be retained in the mouth. In addition to this, it could not be kept clean, and soon became intolerably offensive.

He also had a valve arrangement connected with his plates, but this does not appear in his patent.

JONATHAN DODGE, M. D., New York, March 4, 1843.

This, the inventor styled "the application of the attraction of cohesion, and of capillary attraction in one and the same base, for inserting teeth." A plate was struck up in the usual manner, to fit the gum accurately: this was held to its place by

"cohesive attraction;" being perforated with numerous small holes, brought "capillary attraction" to its aid. A plate was fitted over this, and soldered to it, and to which the teeth were attached, "leaving no space, air-chamber or cavity, in which might be lodged food, mucous, or other matter."

It answered a very good purpose, but never was extended much beyond the inventor's own practice. I dont recollect of seeing any mention of it in any journal.

LEVI GILBERT, New Haven, Conn. February, 1848.

*Claim.*—"My invention is the application of atmospheric pressure to plates used in dentistry: the plate being single, and a chamber being sunk in the central part of the upper surface of the plate, in which a vacuum can be formed by the tongue."

When Mr. Gilbert's patent was announced, it was met, north, east, west and south, by the assertion that the same object had been accomplished long before, and in the same manner, and Mr. G. soon abandoned all hope of pecuniary emolument, and we believe, has given his invention to the public.

JOHN A. CLEAVELAND, Charleston, S. C., June 20, 1850.

In this, a plate is struck up to the gum, and in the central part of the plate an opening is made of about the size, and in the same position as the chamber in Gilbert's. Another plate is fitted over this, meeting it on the alveolar ridge, and separated from it in the palatine vault one-eighth of an inch, more or less, and soldered to it. He claims that space *between* these two plates, as his invention, and not that part between the external plate and gum, at the point where the opening is made in the internal plate. If he can distinguish the difference in the principle between his plate and Rigg's, he can do more than I can, and why a patent should be granted two things so nearly identical, is a question which the commissioner might answer.

